# Non-occlusive mesenteric ischemia associated with enteral feeding after esophagectomy for esophageal cancer: report of two cases and review of the literature

**DOI:** 10.1186/s40792-019-0580-2

**Published:** 2019-02-20

**Authors:** Daisuke Kurita, Takeo Fujita, Yasumasa Horikiri, Takuji Sato, Hisashi Fujiwara, Hiroyuki Daiko

**Affiliations:** Division of Esophageal Surgery, National Cancer Center Hospital East, 6-5-1 Kashiwanoha, Kashiwa, Chiba 277-8577 Japan

**Keywords:** Non-occlusive mesenteric ischemia, Enteral feeding, Esophagectomy

## Abstract

**Background:**

Non-occlusive mesenteric ischemia (NOMI) is a rare but life-threatening complication of early postoperative enteral feeding. We herein report two patients who developed NOMI during enteral feeding after esophagectomy.

**Case presentation:**

In case 1, a 75-year-old man with no medical history was diagnosed with multiple primary cancers of the esophagus, stomach, and kidney. He underwent percutaneous endoscopic gastrostomy tube placement followed by thoracoscopic esophagectomy and cervical esophagostomy placement as the first-stage operation. Gastrostomy feeding was started on postoperative day (POD) 3 with a polymeric formula (ENSURE H®). On POD 7, he developed acute abdominal pain and distension with bloody drainage through the gastrostomy tube. Dynamic computed tomography showed massive hepatic portal venous gas and pneumatosis intestinalis. Angiography showed diffuse spasms in the branches of the superior mesenteric artery. Under a diagnosis of NOMI, we started intra-arterial infusion of papaverine and prostaglandin E1. His symptoms improved, and he was discharged on POD 48.

In case 2, a 68-year-old man with diabetes and atrial fibrillation was diagnosed with esophageal cancer. His medical history was significant for pylorus-preserving gastrectomy for gastric cancer and small bowel resection for trauma. He underwent thoracoscopic esophagectomy, open total gastrectomy, colonic reconstruction, and jejunostomy tube placement. Adhesiolysis for abdominal severe adhesions caused by previous operations was difficult. Jejunostomy feeding was started on POD 3 with a polymeric formula (Racol®). On POD 7, he developed persistent diarrhea and cervical anastomotic leakage. On POD 9, he developed acute abdominal pain and distension with bloody drainage through the jejunostomy tube. Dynamic computed tomography showed the same findings as in case 1. Under a diagnosis of NOMI, we started intravenous infusion of papaverine and prostaglandin E1. His symptoms improved, and he was discharged on POD 28.

**Conclusions:**

The causes of feeding-related NOMI may include the use of a high-osmolarity formula, preoperative malnutrition, abdominal adhesiolysis, systemic inflammation after anastomotic leakage, and a medical history of diabetes and atrial fibrillation. NOMI should be considered as a differential diagnosis in patients with these risk factors and clinical features such as acute abdominal pain and distension during enteral feeding.

## Background

Previous reports have shown that early postoperative enteral nutrition is preferred over parenteral nutrition for patients undergoing esophagectomy because it conserves gut integrity and improves immunological function with a reduction in infectious complications [1, 2]. Thus, early postoperative enteral feeding has become a routine method in many institutions. However, enteral feeding is commonly associated with mild gastrointestinal discomfort and occasionally with severe complications [2]. In particular, non-occlusive mesenteric ischemia (NOMI) is a rare but life-threatening complication of enteral feeding [3]. We herein report our experience with two patients who developed NOMI associated with enteral feeding after esophagectomy for esophageal cancer and present a review of the literature.

## Case presentation

### Case 1

A 75-year-old man with no medical history presented with dysphagia. Upper gastrointestinal endoscopy revealed type III esophageal cancer in the middle thoracic esophagus and type II gastric cancer in the cardia. Computed tomography (CT) showed a left renal tumor and multiple swollen lymph nodes in the neck, mediastinum, and abdomen, including the left renal hilar and para-aortic regions. Thus, he was diagnosed with multiple primary cancers of the esophagus (T3N3M0), stomach (T2N1M0), and kidney (T1bN1M0) according to the Union for International Cancer Control (UICC) 7th edition. He received two cycles of neoadjuvant chemotherapy with nedaplatin and 5-fluorouracil followed by a two-stage operation to decrease the surgical stress. The first-stage operation involved preoperative percutaneous endoscopic gastrostomy tube placement followed by thoracoscopic subtotal esophagectomy in the prone position and cervical esophagostomy placement with mediastinal and cervical lymphadenectomy. The operation was uneventful; it involved 29 mL of blood loss and took 284 min to complete.

**Fig. 1:**
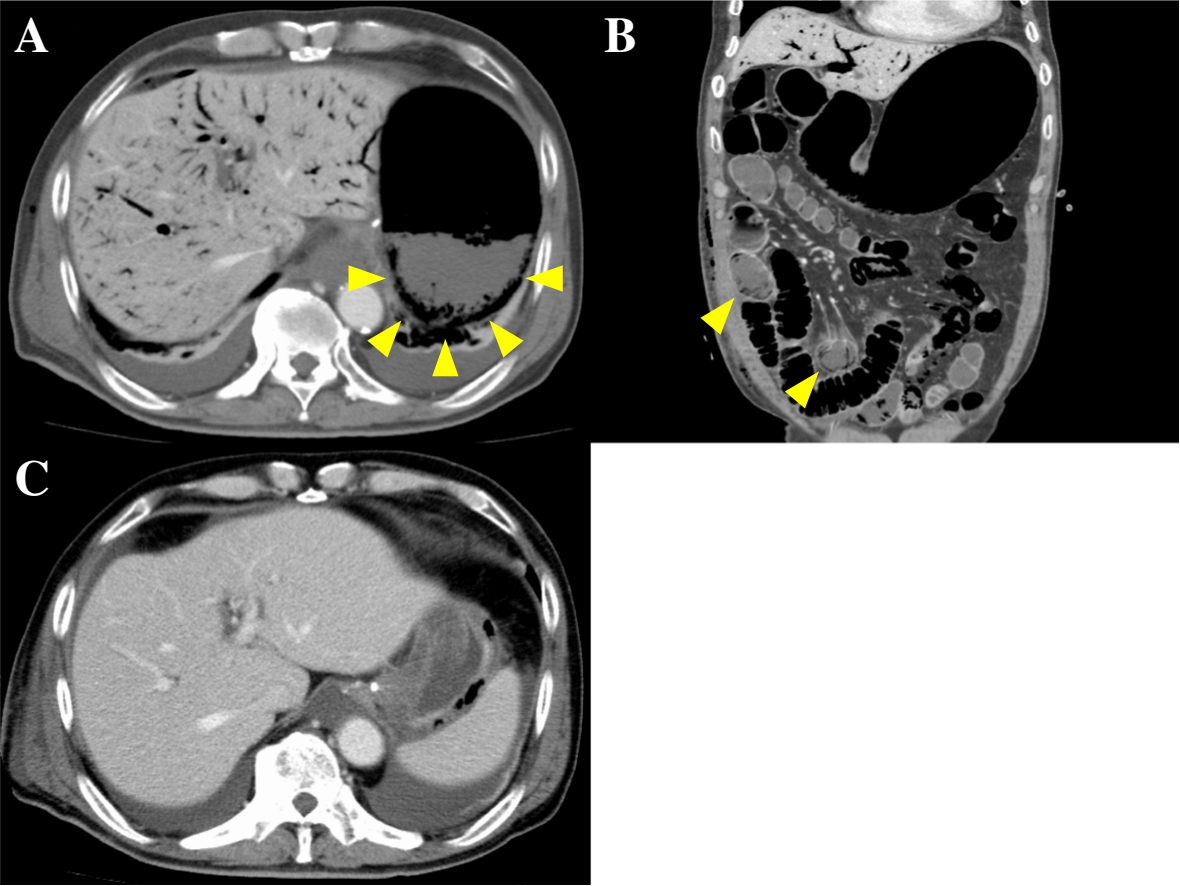
**a**, **b** Computed tomography of the abdomen showing massive hepatic portal venous gas and a dilated gastrointestinal tract with pneumatosis intestinalis (*arrowheads*). **c** Follow-up computed tomography showing a significant reduction of hepatic portal venous gas

**Fig. 2:**
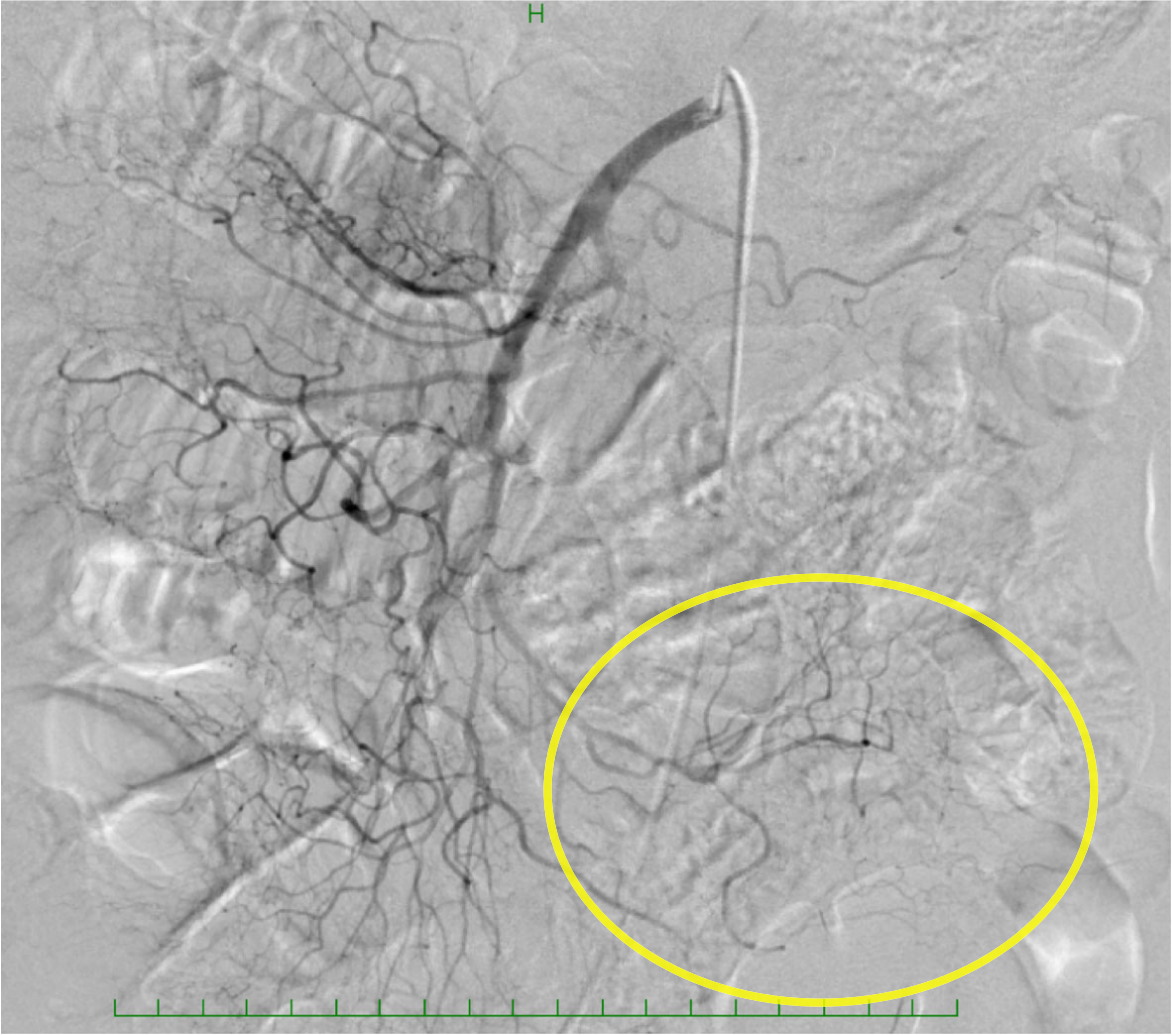
Angiography of the superior mesenteric artery showing diffuse spasms in the branches and poor splanchnic blood flow

Gastrostomy feeding was started on postoperative day (POD) 3 with a polymeric formula (ENSURE H®; 700 mOsm/kg) at a rate of 20 mL/h for 15 h per day, which was increased to 40, 60, and 80 mL/h for 15 h per day on POD 5, 6, and 7, respectively. The postoperative course was uneventful, although the patient developed diarrhea followed by acute abdominal pain and distension with bloody drainage through the gastrostomy tube on POD 7. On examination, he was febrile at 37.2 °C, but the rest of his vital signs were normal. His abdomen was distended with mild diffuse tenderness without guarding or rigidity. Laboratory evaluation revealed an elevated white blood cell count and C-reactive protein level of 11.9 × 10^9^/L and 59.0 mg/L, respectively. Arterial blood gas analysis showed no signs of metabolic acidosis. Dynamic CT showed massive hepatic portal venous gas extending to the superior mesenteric vein, a dilated gastrointestinal tract with pneumatosis intestinalis, segmental poor enhancement of the bowel wall, and small amounts of ascites (Fig. 1a, b). Angiography showed diffuse spasms in the branches of the superior mesenteric artery and poor splanchnic blood flow with no signs of mesenteric arterial thrombosis (Fig. 2). Under a diagnosis of NOMI, we started intra-arterial infusion of papaverine, prostaglandin E1, and heparin through the angiography catheter with intravenous infusion of antibiotics. His symptoms gradually improved, and a CT scan on POD 8 showed a significant reduction of hepatic portal venous gas (Fig. 1c). Gastrostomy feeding was restarted on POD 21, and he was discharged on POD 48. Two months after the first-stage operation, he underwent partial gastrectomy and reconstruction with a gastric tube without nephrectomy or abdominal lymph node dissection because of his poor general condition. He died of systemic metastasis 9 months after the first operation.

### Case 2

A 68-year-old man with diabetes and atrial fibrillation presented with dysphagia. His medical history was significant for pylorus-preserving gastrectomy for gastric cancer and small bowel resection for trauma. Upper gastrointestinal endoscopy revealed type II esophageal cancer in the lower thoracic esophagus. CT showed multiple swollen lymph nodes in the neck, mediastinum, and abdomen. Thus, he was diagnosed with esophageal cancer (T3N3M0) according to the UICC 7th edition. He underwent three cycles of neoadjuvant chemotherapy with docetaxel, cisplatin, and 5-fluorouracil followed by thoracoscopic subtotal esophagectomy in the prone position, open total gastrectomy, colonic reconstruction, and jejunostomy tube placement with three-field lymphadenectomy. During the operation, adhesiolysis for abdominal severe adhesions caused by previous operations was difficult. The blood loss volume and operation time were 448 mL and 510 min, respectively.

Jejunostomy feeding was started on POD 3 with a polymeric formula (Racol® NF; 400 mOsm/kg) at a rate of 20 mL/h for 15 h per day, which was increased to 40 and 60 mL/h for 15 h per day on POD 4 and 6, respectively. The postoperative course was uneventful, although he developed persistent diarrhea and cervical anastomotic leakage on POD 7. The anastomotic leakage improved with conservative treatment, although he developed severe diarrhea followed by acute abdominal pain and distension with bloody drainage through the jejunostomy tube on POD 9. On examination, his vital signs were normal, and his abdomen was distended with mild diffuse tenderness without guarding or rigidity. Laboratory evaluation revealed an elevated white blood cell count and C-reactive protein level of 14.3 × 10^9^/L and 26.0 mg/L, respectively. Arterial blood gas analysis showed no signs of metabolic acidosis. Dynamic CT showed the same findings as in case 1. Under a diagnosis of NOMI, we started intravenous infusion of papaverine, prostaglandin E1, and antibiotics. His symptoms gradually improved, and a CT scan on POD 10 showed a significant reduction of hepatic portal venous gas. He started oral intake on POD 21 and was discharged on POD 28. He was alive without recurrence 9 months after the operation.

## Discussion

NOMI is a rare but life-threatening complication of early postoperative enteral feeding. The reported incidence of feeding-related bowel necrosis, which is most likely to be caused by NOMI, varies from 1.2 to 1.7% [3, 4]. The pathogenesis and clinical features of NOMI remain poorly understood because of the rarity of this disease.

The pathogenesis of NOMI can be explained by non-occlusive reduction of arterial blood flow, most commonly due to primary splanchnic vasoconstriction [5]. Furthermore, the known risk factors for NOMI include cardiovascular disease (heart failure, aortic insufficiency, arrhythmia, and arteriosclerosis), hypovolemic dynamics (dehydration and bleeding), sepsis, dialysis, and administration of vasoconstrictive medications [6, 7]. However, its association with enteral feeding remains unclear and may be multifactorial. Previous reports have suggested that the mechanism of feeding-related NOMI is as follows. First, the absorption of intraluminal nutrients may adversely increase energy demands in metabolically stressed enterocytes, which promote splanchnic blood flow. In the presence of hypoperfusion or inadequate resuscitation, these increased energy demands in combination with a decreased oxygen supply can worsen mesenteric ischemia. Second, enteral nutrition administered on the background of ileus allows bacterial overgrowth, causing accumulation of intraluminal gas and toxins that injure the mucosa [4, 8].

In case 1, the intestinal accumulation of a high-osmolarity formula may have created an osmotic gradient with a rapid fluid shift into the intestinal lumen, leading to intestinal distension and poor splanchnic blood flow [9]. Furthermore, high concentrations of carbohydrates might have provided substrates for excessive bacterial fermentation, causing intestinal distention and toxic injury [10]. Preoperative gastrointestinal dysfunction caused by poor oral intake and malnutrition due to multiple advanced cancers may have also contributed to the development of NOMI.

In case 2, in addition to the surgical stress of open total gastrectomy and colonic reconstruction, aggressive abdominal adhesiolysis because of previous operations may have induced the patient’s severe postoperative gastrointestinal motility disorder. The development of systemic inflammation after anastomotic leakage may have further suppressed gastrointestinal motility. Finally, the medical history of diabetes and atrial fibrillation might have influenced the development of a poor blood supply.

Including the 2 cases in the current report, 39 cases of feeding-related bowel necrosis or NOMI after surgery for oncologic indications have been reported in the English-language literature (Table 1) [3, 4, 6, 7, 11–20]. These cases included 14 men and 8 women (sex data not reported in 17 cases) with a mean age of 65.6 years (range, 38–85 years). The comorbidities were hypertension in 8 patients, diabetes in 4, atrial fibrillation in 3, myocardial infarction in 1, arteriosclerosis obliterans in 1, and hyperlipidemia in 1; all of these comorbidities are known or suspected risk factors for NOMI. The most common primary surgery was pancreaticoduodenectomy in 14 patients followed by total gastrectomy in 10, esophagectomy in 7, and others in 8 (only jejunostomy tube placement in 2). Enteral feeding was started within 72 h after surgery in most patients. The diagnosis of bowel necrosis or NOMI was made at a median of 6 days (range, 1–17 days) from the start of feeding. The median rate of feeding at the time of diagnosis was 72 mL/h (range, 25–125 mL/h). The median osmolality of feeding was 359 mOsm/kg (range, 300–700 mOsm/kg). Emergency laparotomy was performed in 35 patients, including resection of the necrotic intestine in 24 patients. The surgical findings in these 35 patients were bowel necrosis beginning near the jejunostomy tube insertion site and extending distally in 23 patients (65.7%) and bowel necrosis containing inspissated, semi-solid feeding tube contents in 13 patients (37.1%). These surgical findings indicated enteral feedings as a cause of bowel necrosis or NOMI. The overall mortality rate among the 39 patients was 49%.

**Table 1:** Thirty-nine cases of feeding-related bowel necrosis or NOMI after surgery for oncologic indications

Author	Age	Sex	Comorbidity	Diagnosis	Primary surgery	Start of feeding (POD)	Days on feeding	Osmolality of feeding (mOsm/kg)	Maximum rate (ml/h)	Procedure	Surgical findings	Outcome
Zern [11]	81	F	ND	Ovarian cancer	Hysterectomy, colorectomy	0	6	650	100	None	–	Survival
Brenner [12]	73	M	Hypertension, diabetes	Bladder cancer	Cystectomy	1	10	ND	75	None	–	Death
Schunn [13]	38	M	ND	Gastric cancer	Total gastrectomy	2	4	490	40	Bowel resection	Entire small bowel necrosis with inspissated tube feeds	Survival
	61	F	None	Gastric cancer	Distal gastrectomy	0	9	367	60	Bowel resection	Bowel necrosis distal to the jejunostomy with inspissated tube feeds	Survival
	71	F	Hypertension	Pancreatic cancer	Pancreaticoduodenectomy	2	7	310	70	Bowel resection	Bowel necrosis distal to the jejunostomy	Death
	85	M	None	Pancreatic cancer	Jejunostomy	2	4	300	60	Bowel resection	Bowel necrosis distal to the jejunostomy	Death
Rai [14]	58	F	ND	Pancreatic cancer	Pancreaticoduodenectomy	1	14	300	100	Laparotomy	Bowel necrosis distal to the jejunostomy with inspissated tube feeds	Death
Lawlor [6]	57	M	ND	Esophageal cancer	Esophagectomy	1	3	375	100	Bowel resection	Bowel necrosis distal to the jejunostomy	Survival
	71	F	ND	Colon cancer	Gastrojejunostomy	2	6	300	50	Bowel resection	Bowel necrosis distal to the jejunostomy	Survival
Jorba [7]	70	M	ND	Cholangiocarcinoma	Pancreaticoduodenectomy	1	6	ND	ND	Bowel resection	Bowel necrosis distal to the jejunostomy with inspissated tube feeds	Death
Messiner [15]	52	ND	ND	Pancreatic cancer	Pancreaticoduodenectomy	ND	ND	ND	ND	Bowel resection	Bowel necrosis with inspissated tube feeds	Survival
	71	ND	ND	Pancreatic cancer	Pancreaticoduodenectomy	ND	ND	ND	ND	Laparotomy	Bowel necrosis with inspissated tube feeds	Death
	79	ND	ND	Ampullary cancer	Pancreaticoduodenectomy	ND	ND	ND	ND	Laparotomy	Bowel necrosis with inspissated tube feeds	Death
Thaler [16]	74	M	ND	Pancreatic cancer	Pancreaticoduodenectomy	2	4	ND	ND	Bowel resection	Bowel necrosis distal to the jejunostomy	Survival
Melis [17]	54	F	Hypertension	Esophageal cancer	Esophagectomy	1	6	460	70	Bowel resection	Bowel necrosis distal to the jejunostomy	Death
Spalding [4]	76	M	Atrial fibrillation	Gastric cancer	Total gastrectomy	1	3	300	125	Laparotomy	Proximal jejunum necrosis	Death
	65	F	None	Gastric cancer	Total gastrectomy	1	4	300	75	Laparotomy	Entire small bowel necrosis	Death
	55	M	None	Gastric lymphoma	Total gastrectomy	1	17	300	125	Bowel resection	Entire small bowel necrosis	Death
	72	M	None	Ampullary cancer	Pancreaticoduodenectomy	1	4	300	75	Laparotomy	Entire small bowel and right colon necrosis	Death
	39	M	None	IPMN	Pancreaticoduodenectomy	1	6	300	125	Bowel resection	Partial small bowel ischemia	Survival
	55	F	None	Pancreatic cancer	Pancreaticoduodenectomy	1	12	460	85	Laparotomy	Entire small bowel necrosis	Death
Sarap [18]	85	M	Hypertension	Gastric cancer	Distal gastrectomy	1	2	ND	25	Laparotomy	Entire small bowel and right colon necrosis	Death
Qureshi [19]	58	M	Myocardial infarction	Esophageal cancer	Esophagectomy	2	1	ND	ND	Laparotomy	Entire small and large bowel necrosis	Death
Al-Taan [3]	ND	ND	ND	Gastric cancer	Total gastrectomy	1	13	ND	50	Bowel resection	Bowel necrosis distal to the jejunostomy with inspissated tube feeds	Survival
	ND	ND	ND	Gastric cancer	Total gastrectomy	1	7	ND	55	Bowel resection	Bowel necrosis distal to the jejunostomy with inspissated tube feeds	Death
	ND	ND	ND	Gastric cancer	Total gastrectomy	1	6	ND	50	Bowel resection	Bowel necrosis distal to the jejunostomy with inspissated tube feeds	Survival
	ND	ND	ND	Gastric cancer	Total gastrectomy	1	7	ND	52	Laparotomy	Bowel necrosis distal to the jejunostomy with inspissated tube feeds	Survival
	ND	ND	ND	Gastric cancer	Total gastrectomy	1	5	ND	50	Bowel resection	Bowel necrosis distal to the jejunostomy with inspissated tube feeds	Survival
	ND	ND	ND	Gastric cancer	Total gastrectomy	1	4	ND	50	Bowel resection	Bowel necrosis distal to the jejunostomy with inspissated tube feeds	Survival
Sethuraman [20]	70	ND	None	Pancreatic cancer	Pancreaticoduodenectomy	ND	ND	ND	ND	Bowel resection	Bowel necrosis distal to the jejunostomy	Death
	71	ND	Hypertension	Insulinoma	Total pancreatectomy	22	3	375	ND	Bowel resection	Bowel necrosis distal to the jejunostomy	Death
	72	ND	Atrial fibrillation	Ampullary cancer	Pancreaticoduodenectomy	3	2	620	ND	Bowel resection	Bowel necrosis distal to the jejunostomy	Survival
	50	ND	Arteriosclerosis obliterans	Pancreatic cancer	Total pancreatectomy	3	9	600	ND	Bowel resection	Bowel necrosis distal to the jejunostomy	Survival
	69	ND	Hypertension	Pancreatic cancer	Pancreaticoduodenectomy	3	10	620	ND	Bowel resection	Bowel necrosis distal to the jejunostomy	Death
	60	ND	Hyperlipidemia	Esophageal cancer	Esophagectomy	3	3	350	ND	Bowel resection	Bowel necrosis distal to the jejunostomy	Survival
	55	ND	Hypertension, diabetes	Pancreatic cancer	Pancreaticoduodenectomy	3	5	350	ND	Bowel resection	Bowel necrosis distal to the jejunostomy	Survival
	74	ND	Hypertension, diabetes	Esophageal cancer	Esophagectomy	2	14	350	ND	Bowel resection	Bowel necrosis distal to the jejunostomy	Survival
Our data	75	M	None	Esophageal cancer	Esophagectomy	3	4	700	80	Papaverine	–	Survival
	68	M	Diabetes, atrial fibrillation	Esophageal cancer	Esophagectomy	3	6	400	60	Papaverine	–	Survival

*ND* no data, *M* man, *F* female, *IPMN* intraductal papillary mucinous neoplasm

*Clostridium difficile* infection has been reported as a risk factor for developing feeding-related bowel necrosis [20], although a screening test was performed in only one of 39 cases and was negative [13]. In addition, blood culture was collected in two cases, one of them detecting *Klebsiella pneumoniae* and *Streptococcus viridans*, suggesting intestinal infection and bacterial translocation were associated with the causes of bowel necrosis [12, 13]. However, in this case, small intestinal bacterial overgrowth was possibly caused by the accumulation of enteral feeding as mentioned above.

With regard to the clinical presentation of NOMI in our patients, enteral feeding was started at a low rate and slowly increased while paying attention to the development of feeding intolerance, although acute abdominal pain and distension with bloody drainage through the feeding tube occurred without warning. However, because the persistent diarrhea in case 2 suggested the possibility of a gastrointestinal motility disorder, we should have decreased the infusion rate or ceased the feeding. Furthermore, early diagnosis is essential to prevent bowel necrosis and perforation, although it may be difficult for two reasons. First, gastrointestinal discomfort such as mild abdominal pain and distension are relatively common symptoms in patients undergoing enteral feeding after esophagectomy [21]. Second, vital signs are occasionally within normal limits in the early stage of NOMI, as in our patients.

In terms of the therapeutic approach to NOMI in our patients, conservative management was chosen because of the stable vital signs and lack of acute peritoneal signs, and the patients exhibited good recovery. The CT finding of portal venous gas has been suggested to be associated with NOMI in postoperative patients [22], although it does not necessarily indicate the presence of bowel necrosis requiring reoperation. However, for patients suspected to have bowel necrosis or perforation based upon the presence of worsening clinical parameters, early surgical exploration with segmental bowel resection should be considered. According to the American Gastroenterological Association guidelines, the presence of persistent peritoneal signs is an indicator of surgical treatment [23], although we must pay close attention to patients with more severe physical symptoms associated with old age, comorbidities (diabetes, dialysis), and the use of medications (analgesics, sedatives). Furthermore, when deciding whether to continue or stop conservative treatment, a close follow-up CT scan is suggested to be useful because the CT findings 1 day after the onset of NOMI in our patients showed a significant reduction of hepatic portal venous gas. With respect to surgical treatment, it was difficult to determine an appropriate resection line based on surgical findings such as intestinal color, arterial pulsation, and peristalsis [24]. The effect of intraoperative evaluation with indocyanine green fluorescence was recently reported but remains controversial [25]. Therefore, a second-look operation is usually recommended to avoid progressive intestinal ischemia [26].

## Conclusions

To the best of our knowledge, this is the fifth report of feeding-related NOMI after esophagectomy for esophageal cancer. The clinical course outlined in this case report suggests that the causes of feeding-related NOMI may be multifactorial and include the use of a high-osmolarity formula, preoperative malnutrition, abdominal adhesiolysis, systemic inflammation after anastomotic leakage, and a medical history of diabetes and atrial fibrillation. For patients with these risk factors and clinical features such as acute abdominal pain and distension during enteral feeding, NOMI should be considered as a differential diagnosis. Future reports will help to determine the most appropriate management of enteral feeding after esophagectomy.

## Abbreviations

CT: Computed tomography
NOMI: Non-occlusive mesenteric ischemia
POD: Postoperative day
UICC: Union for International Cancer Control
